# Shape Measurement of Single Gold Nanorods in Water Using Open-Access Optical Microcavities

**DOI:** 10.1021/acs.jpclett.4c02104

**Published:** 2024-11-27

**Authors:** Yumeng Yin, Aurélien A. P. Trichet, Jiangrui Qian, Jason M. Smith

**Affiliations:** Department of Materials, University of Oxford, 16 Parks Road, Oxford OX1 3PH, United Kingdom

## Abstract

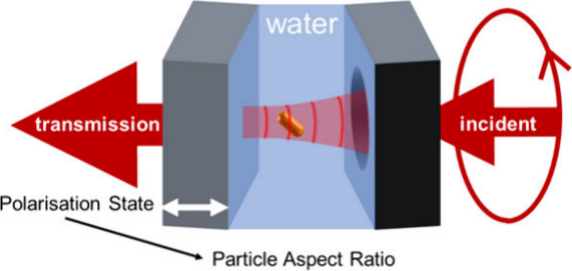

Shape measurement of single nanoparticles in fluids is an outstanding challenge with applications in characterizing synthetic functional nanoparticles and in early warning detection of rod-shaped pathogens in water supplies. Here we introduce a novel technique to measure the aspect ratio of rod-shaped particles by analyzing changes in the polarization state of a laser beam transmitted through an optical microcavity through which the particle diffuses. The resolution in aspect ratio measurement is found to be around 1%. Our work opens the new possibility of in situ and single-particle shape measurement, which has promising applications in nanoparticle characterization, water monitoring, and beyond.

Nanoparticles have in the past 20 years become vital tools in various fields, such as drug delivery^1−3^ and catalysis.^4−6^ The shape of a nanoparticle can significantly influence its properties and performance across these application domains. For example, in nanomedicine, rod-shaped particles allow for a higher drug load owing to their larger surface area to volume ratio^7^ and they can also be advantageous for targeting specific tissues or sites.^8^ The characterization of particle shape is, therefore, an important capability for research and development, and for quality control in manufacturing.

Many natural pathogens, such as *Legionella* and *Escherichia coli* (*E. coli*) bacteria, are also rod-shaped, and the ability to identify their presence in water supplies is important for public health. Shape measurement can therefore provide an early indication of these harmful pathogens in fluids and helps avoid the infection and spreading of diseases.

Various approaches have been taken to measuring nanoparticle shape. If the particles can be isolated and a suitable sample preparation is carried out, direct imaging can be achieved with electron microscopy with resolution at the nanometre-scale or higher. Fluid-based measurements of particle shape are more challenging due to rapid Brownian diffusion, which introduces a random walk to both the location and the orientation of the particles, typically on submillisecond time scales. For larger particles in the micrometre size regime, machine learning algorithms have been used for the shape-based classification of microparticles with moment-based optical holography.^9^ However, the minimum size of particles for which shape can be measured is typically limited by the pixel size and speed of the camera.

Other techniques infer shape from the anisotropic dielectric properties of the particles. The shape dependence of these properties is dependent on the material from which the particle is made. For example, metal nanorods display a plasmon resonance, the frequency of which depends on the aspect ratio. The nanorod displays strong optical absorption at the resonant frequency which can be measured by UV–vis spectroscopy.^10^ Dielectric particles, on the other hand, display no such resonant absorption but have an anisotropic dielectric polarizability that depends weakly on shape and leads to optical light scattering and phase shifts that are dependent on particle orientation in an optical field.^11−13^ Optical scattering techniques such as dynamic light scattering (DLS) and nanoparticle tracking analysis (NTA) are widely available and are in principle sensitive to shape anisotropy. Advanced depolarized DLS,^14^ provides shape information but requires particle concentrations of 10^12^ ml^–1^ for a typical commercial instrument and practically can only obtain averaged values for all the particles in fluids. NTA measures single particles but as an imaging technique, its sampling rate is generally too slow to capture rotational motion, and so far it has not been successful in providing quantitative shape measurement.

The characterization of particle anisotropy by monitoring diffusion dynamics in more detail has also been explored. Simple direct digital video microscopy imaging^15,16^ allows the study of two-dimensional translational diffusion from which shape information can be inferred. In one study, a gold nanorod with a diameter of 84 nm and a length of 164 nm was trapped and rotated with a circularly polarized light to study the hot Brownian motion in a plane.^17^ Three-dimensional diffusion has also been investigated in a viscous environment.^18,19^ Rotational diffusion can also be monitored. For example, single particle orientation and rotation tracking (SPORT) combines traditional single particle tracking (SPT) and Nomarski-type differential interference contrast (DIC),^20^ and permits the systematic study of nanoscale rotational dynamics. In one report, a gold nanorod with a diameter of 40 nm and a length of 118 nm was studied and 2 ms temporal resolution was achieved with gold nanorods on synthetic lipid bilayers.^21^ With the assistance of a convolutional neural network, SPORT has also been used to track anisotropic gold nanoparticle probes in living cells.^20^ In all of these diffusion-based methods, extraction of shape information is challenging as the dynamics depend heavily on the local environment and often occur on faster time-scales than are readily accessible by imaging systems. To capture quicker translational and rotational diffusion of smaller nanorods, more advanced methods must be put forward.

Optical microcavities provide a means to create a small, highly resonant optical mode sensitive to small perturbations and have been used previously to measure the polarizability and hydrodynamic diameter of individual spherical nanoparticles in a fluid medium.^22^ In this work, we show that a similar measurement can be performed on single nanorods to determine their aspect ratio by measuring induced changes to the polarization state of a probe laser beam transmitted through the microcavity. Each individual measurement is performed in a few microseconds and repeated every 100 μs, allowing rotational and translational diffusion of small particles to be captured. The aspect ratio measurements obtained using this method are compared with those from scanning electron microscopy (SEM).

Figure 1a depicts the measurement principle. The microcavity consists of opposing concave and planar mirrors, fabricated using low-loss distributed Bragg reflector (DBR) coatings (see SI, section 1), to form resonant optical modes.^23,24^Figure 1b shows a scanning electron microscopy (SEM) image of an array of concave micromirrors fabricated with different radii of curvature (RoC). In this work, a microcavity was formed using a concave mirror with a RoC of 25 μm and depth of 600 nm, positioned opposite a planar mirror to establish a cavity length of 1.5 μm (Figure 1a). The measurement is based on the change in polarization state of a laser beam transmitted through the cavity. The laser beam has a fixed wavelength (λ = 640 nm) and is circularly polarized to excite the orthogonal linear polarization states of the cavity mode equally. The cavity mode wavelength is swept through resonance with the laser wavelength by modulating the cavity length sinusoidally at a frequency of 5 kHz using a piezoelectric actuator. The intensity and polarization of the transmitted light are measured in the time domain with 10 MHz bandwidth, using an avalanche photodiode (APD) and Stokes analyzer with balanced photodiodes,^25^ respectively (Figure 1c and SI, sections 1 and 2). The data are then converted from the time domain to the frequency domain (SI, section 3) to reveal the mode spectra for analysis. In the absence of a nanoparticle the cavity resonance has a spectral line width of about 30 GHz, corresponding to a *Q*-factor of around 16000.

**Figure 1:**
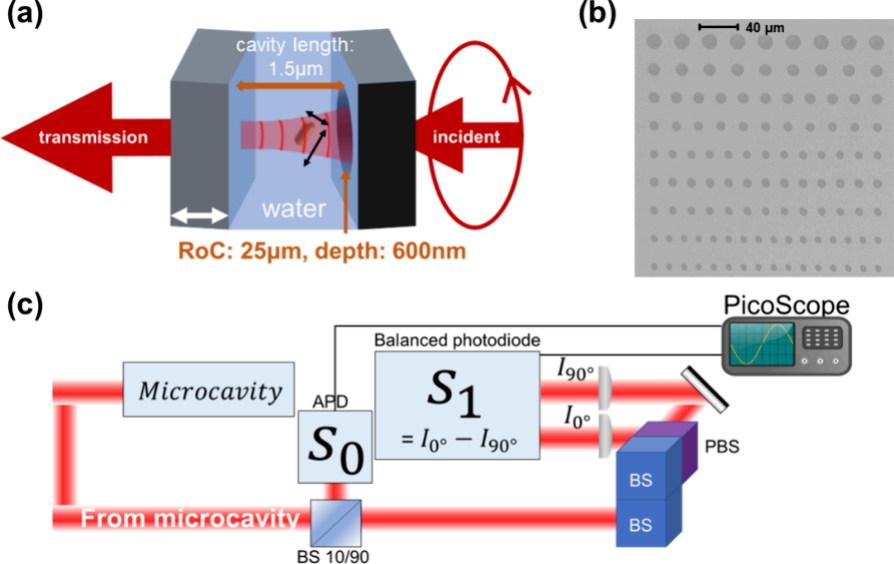
Experimental apparatus. (a) Schematic diagram of a gold nanorod diffusing in the microcavity. Double-headed black arrows indicate directions parallel and transverse to the rod axis. The double-headed white arrow indicates the scanning of the planar mirror to modulate the cavity length. (b) SEM image of an array of micromirrors of different sizes. In this work a micromirror with RoC = 25 μm, from the uppermost row of the array in the image, was used for cavity construction. (c) Schematic diagram of the apparatus used to analyze the transmitted light. *S*_0_ and *S*_1_ refer to the Stokes parameters measured (eqs 1a and 1b).

Gold nanorods are selected with plasmon resonance at around 550 nm such that the probe wavelength is substantially red-shifted from the resonant wavelength and the coupling to the cavity mode is in the perturbative regime of cavity quantum electrodynamics. When a particle enters the microcavity, the polarizability of the particle relative to the water medium causes a red shift and broadening of the cavity mode, the latter due to a combination of optical absorption and scattering. Since the dielectric polarizability of the nanorod is anisotropic, it splits the cavity mode into two eigenstates with optical polarization parallel to the principal axes of the rod projected onto the cavity plane (*X* and *Y* in Figure 2a). These two orthogonal eigenstates induce different phase shifts in the transmitted beam, such that it obtains a degree of linear polarization which is a function of detuning. Each sweep of the cavity length therefore generates a spectrum for each measured polarization state from which the relevant parameters can be extracted.

**Figure 2:**
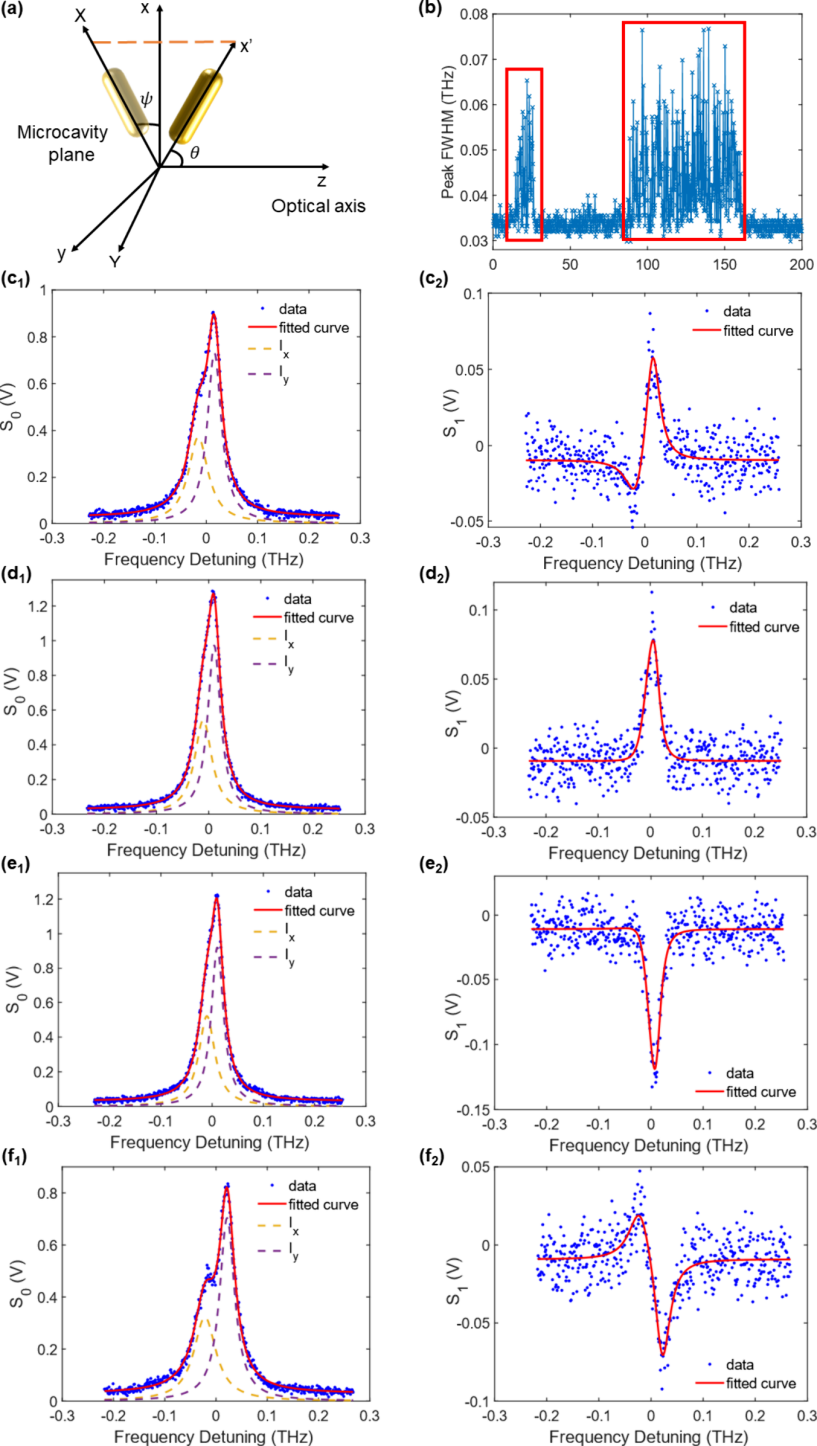
Single-particle detection and raw data. (a) Schematic of the coordinate system used for a nanorod in the microcavity. (b) FWHM of the *S*_0_(Δ) peak monitored over 200 ms after injecting the sample A solution into the flow cell. The data show two discrete single-particle events. (c–f) Examples of individual *S*_0_(Δ) and *S*_1_(Δ) peaks during the second event. Blue dots are raw data and red curves are fitted curves using eqs 1a and 1b.

The Stokes parameters *S*_0_ and *S*_1_ in the presence of an anisotropic particle can be expressed as functions of detuning, Δ, relative to the resonant frequency of the microcavity in the presence of a spherical particle with the same volume in eqs 1a and 1b (see SI, section 4 for details):
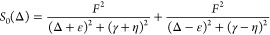
1a
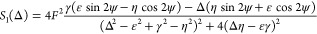
1bwhere *F* is the amplitude of the incident field and γ is the total photon loss rate from the cavity in the presence of the spherical particle. 2ε and 2η represent the particle anisotropy and are the respective differences in the resonant frequency and in the loss between the two orthogonal cavity eigenstates. ψ is the azimuthal angle of the nanorod about the optical axis of the cavity as referred to the instrument axes (Figure 2a).

*S*_1_ provides the more sensitive measurement of the particle anisotropy since its amplitude is proportional to ε and η, but data analysis is complicated by the dependence on ψ. Alternatively, if the particle is sufficiently anisotropic that the mode splitting is comparable in magnitude to the mode width, ε and η can be established from *S*_0_ alone.

With ε and η determined for a given measurement, the particle aspect ratio is conveniently derived from the “anisotropy loss tangent” . For small particles, in which the scattering albedo (the fraction of the total extinction that is due to scattering) is small, losses (and, therefore, η) are dominated by absorption. In this scenario, Φ is independent of both the position and the orientation of the nanorod within the cavity mode, and is determined entirely by the two complex polarizabilities of the particle in eq 2 (SI, sections 5 and 6):
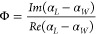
2where α_*W*_ and α_*L*_ are the polarizabilities along the short and long axis of the nanorod, respectively (see SI, section 6 for details). For particles with a substantial scattering albedo, Φ depends on both the orientation and the position of the particle in the microcavity, but shape information can still be deduced with certain assumptions. In either case, the aspect ratio μ is readily deduced from Φ.

To demonstrate the measurement method, gold nanoparticles in aqueous solution of three designs were tested. Samples A and B were nanorods (Nanopartz A12-25-550-CTAB-DIH-1-25 and A12-40-550-CTAB-DIH-1-25, respectively) with a nominal aspect ratio of μ = 1.36 (length/width = 34/25 nm) and μ = 1.50 (length/width = 60/40 nm), respectively. Sample C was a control sample of nanospheres with diameter of 60 nm (Sigma-Aldrich 742015-25 mL). The nanorod suspensions of samples A and B were stabilized using cetyltrimethylammonium bromide (CTAB) as a surfactant while the nanospheres of sample C were stabilized in citrate buffer.

SEM characterization of the nanorod samples was performed to provide independent measurement of the aspect ratios (see SI, Figure S4). The extracted values depend on the assumed distribution of orientations of the nanorods relative to the SEM image plane. Assuming the nanorods lay flat yields values of μ = 1.47 ± 0.10 and 1.48 ± 0.09 for samples A and B, respectively, while assuming random orientation yields respective values of μ = 1.56 ± 0.14 and 1.56 ± 0.18.

Based on the independent SEM measurements, average particle volumes were established and theoretical absorption and scattering cross sections^11−13^ were calculated. The resulting scattering albedo was calculated to be 0.27 for sample A and 0.18 for sample B, suggesting that Φ would be only a weak function of particle position and orientation in both cases.

Figure 2b shows an exemplar time trace of the measured cavity mode width over time as particles are introduced into the microfluidic flow cell. Two well-defined periods of tens of milliseconds are observed (red boxes) in which the mode broadens, indicating discrete single particle detection events. Each event provides multiple measurements of *S*_0_(Δ) and *S*_1_(Δ), with both spectra changing as the particle diffuses in the cavity mode.

When the spherical particles of the control sample C were introduced, clear single particle detection events were observed but *S*_0_(Δ) traces showed no splitting of the resonant peak and the *S*_1_ signal remained zero, confirming that they introduced no measurable anisotropy into the sensor (see SI, section 7). When nanorod samples A and B were injected, however, some of the *S*_0_(Δ) traces in each event show splitting of the cavity mode and *S*_1_(Δ) shows clear features. In the data presented below, we determine ε and η directly from *S*_0_(Δ) and use *S*_1_(Δ) to obtain the azimuthal angle ψ.

Figure 2c–f shows examples of individual fits to *S*_0_(Δ) and *S*_1_(Δ) signals during the second single-particle event in Figure 2b. As the nanorod undergoes rotational diffusion, changes in polar angle θ translate to changes in the magnitude of the splitting of the *S*_0_(Δ) peak and the amplitude of the *S*_1_(Δ) response, while changes in ψ translate to changes only in the shape of the *S*_1_(Δ) response.

The sweep rate for the detuning is of order 100 GHz/μs such that each resonance is measured within about 1 μs. The angular displacement of the nanorods caused by rotational diffusion and by optical torques exerted by the intracavity field are small within this time duration and so the particle can be considered stationary for the purposes of analyzing each peak (see SI, section 8). The expected temperature change on the nanorod surface as a result of optical absorption is estimated to be of order 10 K (see SI, section 9), from which we conclude that particle shape change due to heat is negligible.

Each single particle detection event consists of several hundred individual mode sweeps, each of which in turn provides a measure of ψ and Φ. These multiple independent measurements on each particle provide a basis for statistical analysis. Histograms for ψ and Φ for a single-particle event from each of samples A and B are shown in Figure 3a–d. The ψ distributions are seen to be flat, indicating no preferred orientation of the particles in the lab frame. The Φ distributions in each case show a clear single peak in the range 0.2 < Φ < 0.3. Figure 3e illustrates the theoretical relationship between μ and Φ in the absence of scattering (see SI, section 10). Fitting Gaussian functions (normal distributions) to the Φ histograms and converting the mean and standard error values to aspect ratio results in μ = 2.00 ± 0.02 and 1.87 ± 0.02 for the sample A and B particles, respectively. The magnitudes of the standard errors in μ suggest a basic measurement resolution of around 1%.

**Figure 3:**
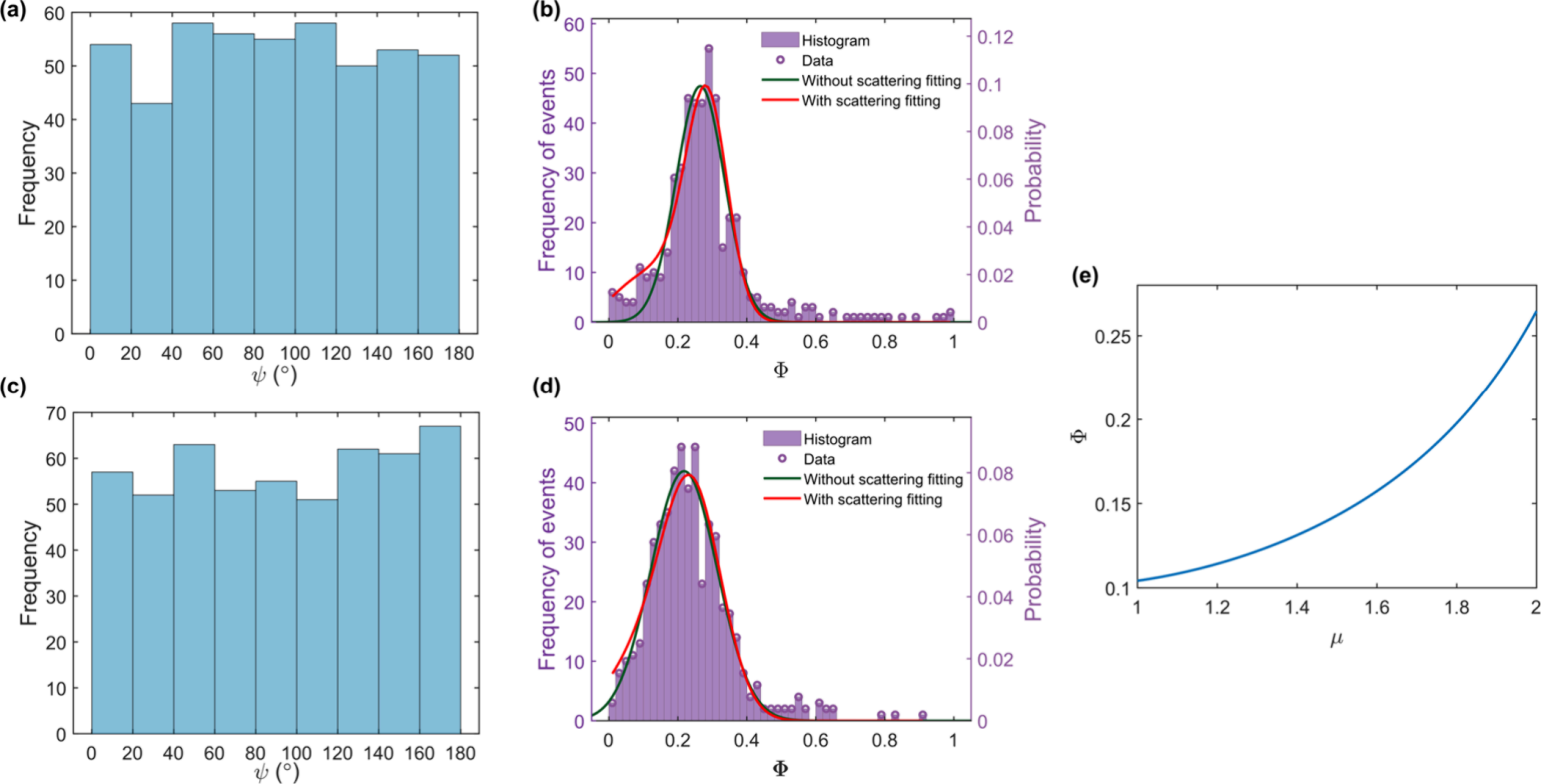
Summary of a single-particle event. Sample A: (a), (b); Sample B: (c), (d). (a, c) Histograms of azimuthal angle ψ. (b, d) Histograms of anisotropy loss tangent Φ. The green curve is the fitting without scattering while the red curve is the fitting with scattering using SEM volume. (e) The numerical relationship between μ and Φ neglecting scattering.

The inclusion of scattering in the theoretical model introduces a dependence of Φ on particle orientation and therefore modifies the expected distribution of Φ values (see SI, section 6). A full analysis of scattering is complicated by the fact that it depends on the optical density of states in the microcavity, which is a function of position within the cavity mode and is beyond the scope of this work: here we assume a uniform optical density of states. Fits using the modified distribution function with a free-space density of states are shown as red solid lines in Figure 3b,d. These fits yield somewhat smaller values for the aspect ratios, μ = 1.66 ± 0.05 and 1.69 ± 0.04 for the two exemplar particles of samples A and B, respectively.

To compare the optical analysis method with SEM, measurements of 30 particles for each of samples A and B were carried out with each technique. The total measurement time for 30 particles with the optical analysis method was around 18 min with the concentrations used, compared with 2 h using the SEM method. The duration of single particle events in Figure 2b suggests that this measurement time could potentially be reduced to ∼10 s. Figure 4 shows a comparison between the results obtained. Figure 4a shows a histogram of the aspect ratios measured using optical analysis and analyzed neglecting scattering effects. The mean values and standard deviations obtained are μ = 1.85 ± 0.13 and 1.89 ± 0.11 for samples A and B, respectively. Figure 4b shows the comparative SEM data, analyzed assuming the nanorods lay flat in the plane of the micrograph, which yields values of 1.47 ± 0.10 and 1.48 ± 0.09 respectively, while values of 1.56 ± 0.17 and 1.57 ± 0.16 are obtained if one assumes that the particles in the images are randomly oriented (SI, section 11). Although the mean values produced by the optical method, neglecting scattering, and the SEM analysis differ, the distributions are similar to samples A and B overlapping in both cases–neither method reports a resolvable difference between the aspect ratios of the two samples. Figure 4c illustrates the comparison between SEM data and optical microcavity sensing method with and without scattering considered.

**Figure 4:**
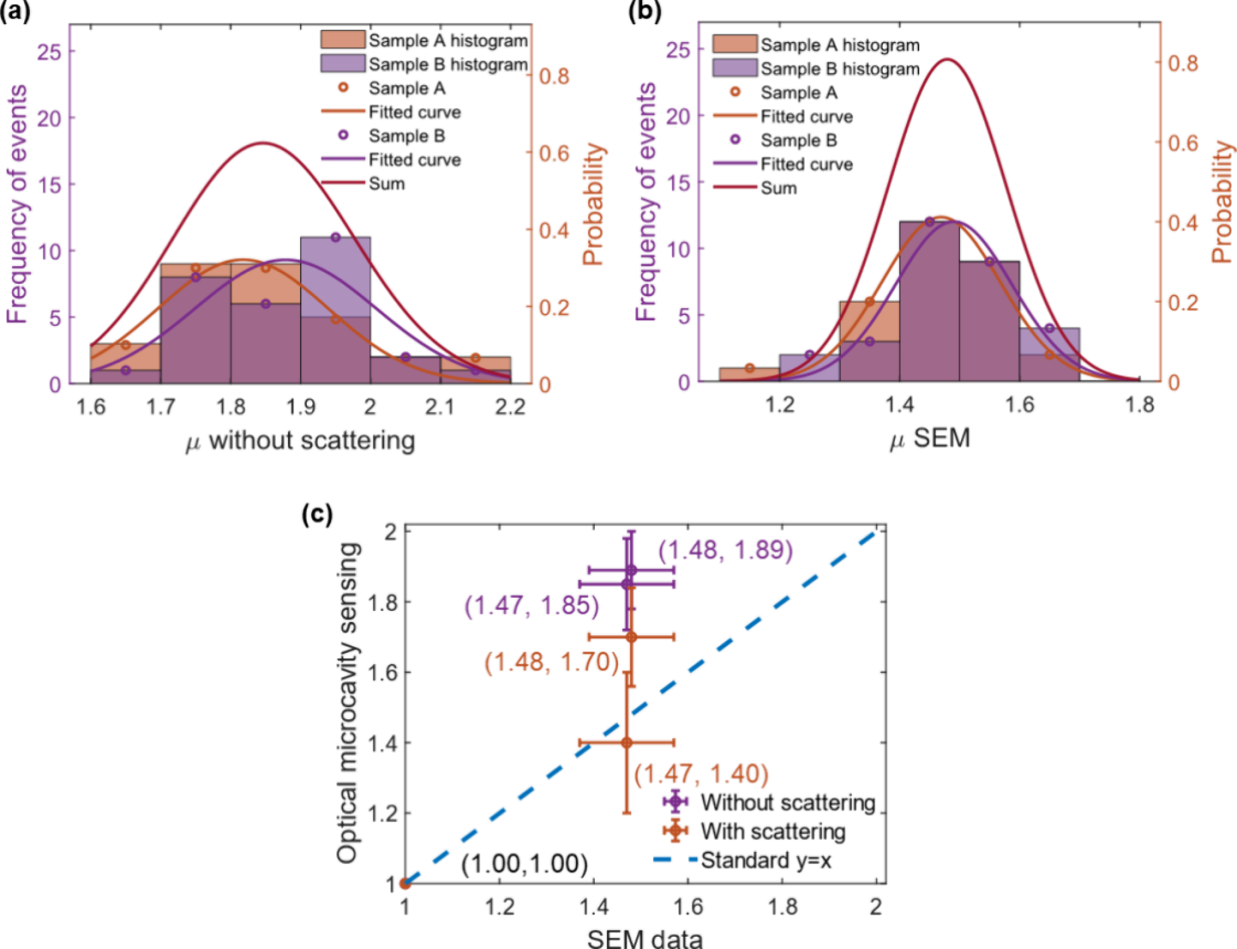
Summary of statistical results of 30 particles from samples A and B. (a) Histograms of aspect ratios measured using optical microcavity sensing neglecting scattering effects. (b) Histograms of aspect ratios measured by SEM assuming the particles lay flat in the SEM images. (c) Relationship between the average aspect ratios measured using optical microcavity sensing with and without scattering and those measured using SEM.

If scattering is included in the analysis and a uniform free-space optical density of states within the cavity is assumed, the aspect ratios of the 30 particles in each sample become μ = 1.40 ± 0.20 and μ = 1.70 ± 0.14, i.e., sample B has a measurably larger average aspect ratio than sample A, in qualitative agreement with the manufacturer’s data. If, further, the average optical density of states in the cavity is reduced to 0.65 times that of the free space,^22^ the calculated aspect ratios become 1.59 ± 0.16 and 1.82 ± 0.12, respectively. Table 1 summarizes the results obtained using the different methods.

**Table 1:** Aspect Ratio Measurements, Comparison between Methods

method	μ of sample A	μ of sample B
manufacturer’s data	1.36	1.50
SEM assuming rods lay flat	1.47 ± 0.10	1.48 ± 0.09
SEM assuming random orientation	1.56 ± 0.14	1.56 ± 0.18
optical microcavity sensing (neglecting scattering)	1.85 ± 0.13	1.89 ± 0.11
optical microcavity sensing (including scattering, )	1.40 ± 0.20	1.70 ± 0.14
optical microcavity sensing (including scattering, )	1.59 ± 0.16	1.82 ± 0.12

One point to note is that the SEM and optical microcavity sensing data are measuring different sets of nanoparticles from the two samples. While there is no obvious reason for selective bias in these measurements, it cannot be discounted entirely. Note also that the volume factors used here to calculate scattering albedo are based on average volume: they can in principle be established on a particle-by-particle basis within the same microcavity analysis apparatus, using either the magnitude of the mode shift^22^ or the temporal autocorrelation function. Indeed these two parameters can be combined to establish the refractive index of the particle at the probe wavelength, removing the need for independent knowledge of the particle composition. Based on the signal-to-noise ratio of the *S*_1_(Δ) data sets in Figure 2, we estimate the minimum aspect ratio that the technique can measure (SI, section 12) to be μ = 1.07.

The estimated accuracy of the technique of 1% is based only on the standard error observed in the evaluation of μ from the multiple data points obtained for each particle. Potential sources of systematic error include the calibration of the frequency axis, which could be minimized by in situ calibration or direct monitoring of the cavity modulation, and approximations made within the theoretical model, in particular where the effects of scattering are considered.

The optical analysis method can be adapted to dielectric nanoparticles such as rod-shaped pathogens. For other complex shapes, not limited to regular shapes such as a rod, like a disk or triangle shape, this technique could still be adapted accordingly to perform more advanced analysis.

In summary, we present a new method utilizing optical microcavities for measuring the aspect ratio of nanorods in solution, and demonstrate its performance on samples of gold nanorods. By measuring Stokes parameters of the transmitted light through the microcavity, the polarizability anisotropy is deduced to obtain the shape anisotropy. Compared with EM, the optical measurement technique is more suitable to perform analysis in solution media, requiring no sample preparation. It can achieve analysis on a single-particle scale, which UV–vis spectrometer and DLS cannot, making it suitable for inline measurements at low particle concentrations. The temporal resolution of 1 μs is fast enough to capture single nanorods’ rotational and translational diffusion in three-dimensional water environment, which overcomes the difficulties faced by techniques such as direct digital video microscopy imaging and SPORT.

## Supporting Information Available

Experimental apparatus (Figure S1); Polarimeter calibration (Figure S2); Conversion between time and frequency domain, Stokes parameters derivation, polarizability anisotropy, anisotropy loss tangent derivation, probability distribution, and spherical particle data (Figure S3); Rotational diffusion analysis (Table S1); Thermal effects, relationship between Φ and μ, SEM results, and analysis (Figures S4 and S5); Minimum aspect ratio analysis (PDF)
