# Supplementary material for: Shape Measurement of Single Gold Nanorods in Water Using Open-Access Optical Microcavities
Source: J Phys Chem Lett. 2024 Nov 27;15(49):12105–11. doi: 10.1021/acs.jpclett.4c02104 (PMC11648073; doi:10.1021/acs.jpclett.4c02104)
Supplement: Supplementary file 1 — jz4c02104_si_001.pdf [file jz4c02104_si_001.pdf]

Shape measurement of single gold nanorods in water using open-access optical microcavities

Supporting Information

Yumeng Yin, Aurélien A. P. Trichet, Jiangrui Qian, Jason M. Smith*

Department of Materials, University of Oxford, 16 Parks Road, OX1 3PH, United Kingdom

Table of contents

1. Experimental Apparatus 2
2. Polarimeter Calibration 3
3. Conversion Between Time and Frequency Domain 3
4. Stokes Parameters Derivation 4
5. Polarisability Anisotropy 5
6. Anisotropy Loss Tangent Derivation and Probability Distribution 5
7. Spherical Particle Data 6
8. Rotational Diffusion Analysis 7
9. Thermal Effects 8
10. Relationship Between Φ and μ 8
11. SEM Results and analysis 8
12. Minimum Aspect Ratio Analysis 9

1. Experimental Apparatus

A complete schematic of the optical apparatus is shown in Figure S1. The intensity of the laser light is adjustable with the combination of a half-wave plate and a polarising beam splitter (PBS). Three parts of the apparatus are identified in blue boxes. Part 1 splits the laser beam into two beams using a half-waveplate and two PBSs. The intensities and alignment of the two beams can be adjusted independently. One beam is the sensing beam to perform the particle characterisation, while the other one is the reference beam to remove background noise. Part 2 of the apparatus includes the microcavity assembly and an imaging system used both for alignment and to perform cavity locking through a proportional-integral-derivative (PID) control system. It provides a locking function by monitoring fringes formed by the transmission of off-resonance LED light through the planar side of the cavity. Part 3 is the polarimeter used to analyse the polarisation state of the transmitted laser light. Within the polarimeter, two 50:50 beam splitters (BS) remove the linear retardance and diattenuation to preserve the original polarisation state of the light. Polarising beam splitters (PBS) split the light into orthogonal components and balanced photodiodes are used to measure intensity differences between them. Picoscope gets triggered by signs of single-particle events and collects the voltage data from the avalanche photodiode (APD) and two balanced photodiodes as a function of time simultaneously for further data analysis. S_3 measures the degree of linear polarisation of the transmitted light. The measurement for S_3 is not illustrated in the main text Fig. 1(c) as the data is not utilised in the extraction of necessary parameters from the model we built.

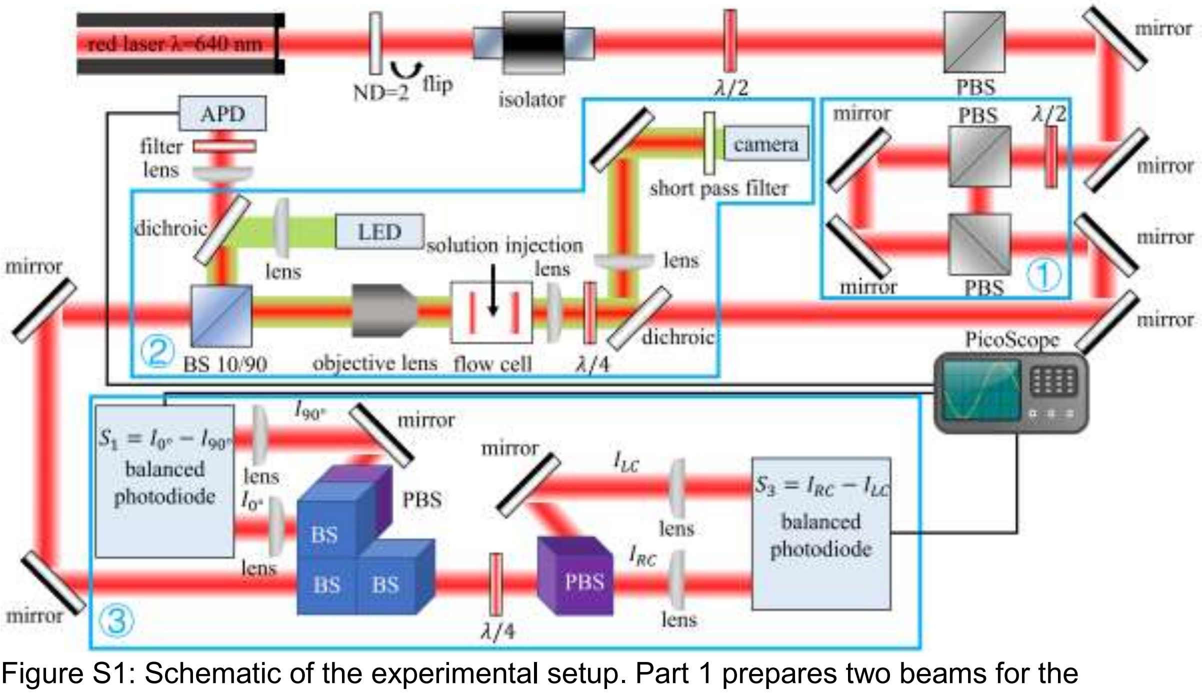

Figure S1: Schematic of the experimental setup. Part 1 prepares two beams for the experiment. Part 2 includes the flow cell and the imaging system for the microcavity array. Part 3 is the polarimeter measuring Stokes parameters.

A microfluidic system was used to inject the solution into the microcavity. Gold nanorods in aqueous solution (Nanopartz Inc.) of two designs (samples A and B) were tested. Sample A (A12-25-550-CTAB-DIH-1-25) has a diameter of 25 nm and length of 34 nm with cetyltrimethylammonium bromide (CTAB) as the stabiliser while sample B(A12-40-550-CTAB-DIH-1-25) has the same absorbed ligand with a diameter of 40 nm and length of 60 nm from supplier's information. Diluted concentrations of around 10^9 nanoparticles /mL provided single particle events every few seconds. Sample volumes of 200-300 μL were

used for each measurement, determined by the dimensions of the flow cell and microfluidic assembly. Upon sample injection, as soon as the first particle was observed entering the cavity, the flow was stopped and the particles in the solution were allowed to diffuse freely within the flow cell reservoir.

Substrates for concave mirrors were prepared using focused ion beam milling. Mirrors were SiO_2/Ta_2O_5 Bragg stacks with reflectivity of 99.99% and 99.92% (Layertec GmbH) for the concave and planar sides respectively.

2. Polarimeter Calibration

Before inserting the flow cell with the cavity, the polarimeter was used to measure Stokes parameters of a prepared laser beam to check its performance. The quarter-waveplate (QWP) in part 2 of the apparatus (Figure S1) was used to prepare the incident light with different polarisation states by rotating at different angles. The Stokes parameters were normalised in equation (1a) and (1b):

$$\widetilde{S_1} = \frac{I_{0^\circ} - I_{90^\circ}}{I_{0^\circ} + I_{90^\circ}} \qquad (1a)$$

$$\widetilde{S_3} = \frac{I_{RC} - I_{LC}}{I_{RC} + I_{LC}} \qquad (1b)$$

These measured values were then compared with the standard theoretical values [1] and the difference was plotted as a function of the QWP rotation angle. Measurements were performed both directly using a power meter and after fibre coupling. The results in Figure S2 show that the measured polarisation state agrees with theoretical prediction to within 5% across all measurements.

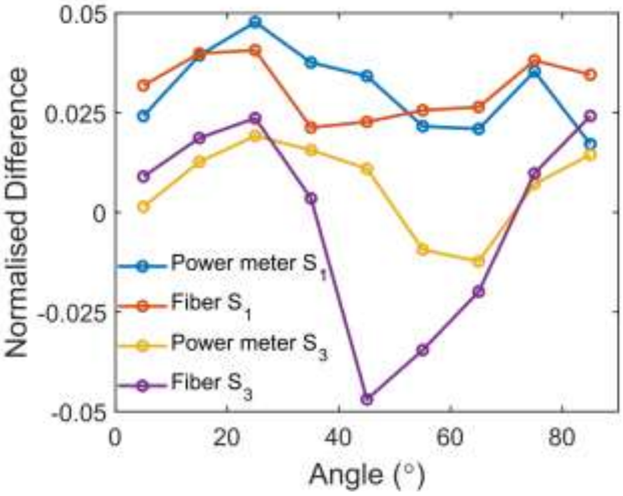

Figure S2: Polarimeter calibration. Normalised Stokes parameters S_1 and S_3 were measured with different rotation angles of the quarter-waveplate. The normalised differences between the measured and theoretical values are plotted.

3. Conversion Between Time and Frequency Domain

With the scanning at 5 kHz, data is recorded in the time domain. It is then converted to the frequency domain as below:

$$w = \frac{c}{\lambda} \qquad (2a)$$

whereby differentiating gives:

$$\frac{\Delta}{w_c} = -\frac{\Delta\lambda}{\lambda} = -\frac{2n_m}{q}\frac{\Delta L}{\lambda} \quad (2b)$$

where Δ is the detuning in units of frequency relative to the midpoint of the two orthogonal modes, w_c is the frequency of the probe laser light, n_m is the refractive index of the liquid medium, q is the mode number, and $\lambda = 640\ nm$ is the wavelength of the probe laser. ΔL is achieved by the sinusoidal scanning of the piezoelectric stacks glued on the planar mirror side. Therefore, the frequency detuning is shown in equation (3):

$$\Delta = -\frac{n_m w_c}{q\lambda} * A_s * [\sin(\omega_s(t - t_0)) - \sin(\omega_s(t_{mid} - t_0))] \quad (3)$$

Where A_s and ω_s are the amplitude and angular frequency of the cavity length modulation, and t_{mid} and t_0 correspond to the times at which the mid-point of the two modes and the mid-point of the modulation are respectively reached. A_s is estimated according to the expected response of the piezoelectric stack to the applied voltage.

4. Stokes Parameters Derivation

The incident light was prepared with a left-handed circular polarisation. The electric fields in the transmitted beam, along the directions parallel (X) and perpendicular (Y) to the major axis of the rod projected into the cavity plane (main text Fig. 2(a)), can then be written as below:

$$e_X = \frac{Fe^{-i\omega t}}{(\Delta + \varepsilon) + i(\gamma + \eta)} \quad (4a)$$

$$e_Y = \frac{iFe^{-i\omega t}}{(\Delta - \varepsilon) + i(\gamma - \eta)} \quad (4b)$$

where F is the frequency of the incident field, Δ represents the detuning between the cavity mode frequency and that of the laser in the presence of a spherical particle, and γ is the total photon loss rate in the cavity in the presence of a spherical particle. 2ε and 2η represent the particle anisotropy and are the differences in the resonant frequency and in the loss between the two orthogonal eigenstates.

Four Stokes parameters are expressed as:

$$S_{00} = |e_X|^2 + |e_Y|^2 \quad (5a)$$

$$S_{10} = |e_X|^2 - |e_Y|^2 \quad (5b)$$

$$S_{20} = \frac{1}{2}|e_X + e_Y|^2 - \frac{1}{2}|e_X - e_Y|^2 \quad (5c)$$

$$S_{30} = \frac{1}{2}|e_X + ie_Y|^2 - \frac{1}{2}|e_X - ie_Y|^2 \quad (5d)$$

whereby combining equations 4 and 5 gives the Stokes parameters referred to the (X,Y) axes. These parameters are then translated to the instrument frame of reference (x, y) (main text Fig. 2(a)), by the axis rotation

$$\begin{pmatrix} S_0 \\ S_1 \\ S_2 \\ S_3 \end{pmatrix} = \begin{pmatrix} 1 & 0 & 0 & 0 \\ 0 & -cos2\psi & sin2\psi & 0 \\ 0 & -sin2\psi & cos2\psi & 0 \\ 0 & 0 & 0 & 1 \end{pmatrix} \begin{pmatrix} S_{00} \\ S_{10} \\ S_{20} \\ S_{30} \end{pmatrix} \quad (6)$$

to give

$$S_0(\Delta) = \frac{F^2}{(\Delta + \varepsilon)^2 + (\gamma + \eta)^2} + \frac{F^2}{(\Delta - \varepsilon)^2 + (\gamma - \eta)^2} \quad (7a)$$

$$S_1(\Delta) = 4F^2 \frac{\gamma(\varepsilon sin2\psi - \eta cos2\psi) - \Delta(\eta sin2\psi + \varepsilon cos2\psi)}{(\Delta^2 - \varepsilon^2 + \gamma^2 - \eta^2)^2 + 4(\Delta\eta - \varepsilon\gamma)^2} \quad (7b)$$

where S_2 and S_3 have been neglected as they are not required in this work.

For the experiment, since the APD and balanced photodiodes (Figure S1) have different responsivities for light intensity, their voltage amplitude varies. However, this can be incorporated into different effective F in the curve fitting process, and won't have any impact on our final results.

5. Polarisability Anisotropy

The anisotropic polarisability of a gold nanorod is described as a complex second-rank tensor. In the nanorod's frame of reference, the tensor takes a diagonal form:

$$\boldsymbol{\alpha} = \begin{pmatrix} \alpha_W & 0 & 0 \\ 0 & \alpha_W & 0 \\ 0 & 0 & \alpha_L \end{pmatrix} \tag{8}$$

where α_W and α_L are the relative polarisabilities along the short and long axis of the nanorod respectively. Gans modification to Mie theory [2], [3], [4] relates these relative polarisabilities to the particle aspect ratio μ as follows:

$$\alpha_{W,L} = \frac{\epsilon - \epsilon_m}{\epsilon_m + p_{W,L}(\epsilon - \epsilon_m)} \tag{9}$$

where

$$p_L = \frac{1}{\mu^2 - 1}\left(\frac{\mu}{2\sqrt{\mu^2 - 1}} \ln\left(\frac{\mu + \sqrt{\mu^2 - 1}}{\mu - \sqrt{\mu^2 - 1}}\right) - 1\right) \quad \text{and} \quad p_W = \frac{1 - p_L}{2}. \tag{10}$$

Here, ϵ is the permittivity of gold [5] at the probe laser wavelength $\lambda = 640\ nm$, ϵ_m is the permittivity of the surrounding medium, and $p_{W,L}$ are the depolarisation factors. The complex nature of ϵ results in $\alpha_{W,L}$ being complex also. Projecting the polarisability tensor onto the (X,Y) plane gives:

$$\boldsymbol{\alpha}' = \begin{pmatrix} \alpha'_L & 0 \\ 0 & \alpha'_W \end{pmatrix} = \begin{pmatrix} \cos^2\theta\, \alpha_W + \sin^2\theta\, \alpha_L & 0 \\ 0 & \alpha_W \end{pmatrix} \tag{11}$$

where θ is the angle of the nanorod to the optical axis (main text Fig. 2(a)). α'_L and α'_W are the polarisabilities along X and Y axis respectively (main text Fig. 2(a)). The real and imaginary parts of $\boldsymbol{\alpha}'$ result in shifts in the mode position and increases in mode width respectively.

6. Anisotropy Loss Tangent Derivation and Probability Distribution

The anisotropy loss tangent is defined as $\Phi = \frac{\eta}{\varepsilon}$ in the main text:

$$\Phi = \frac{\eta}{\varepsilon} = \frac{\frac{w_c(\Delta\alpha_i + \Delta_{scat})}{4V_m} I(\vec{r})}{\frac{w_c \Delta\alpha_r}{4V_m} I(\vec{r})} \tag{12}$$

where w_c is the frequency of the probe laser, V_m is the mode volume, $I(\vec{r})$ is the relative mode intensity at the particle position normalised to the strongest intensity in the cavity, $\Delta\alpha_i$, $\Delta\alpha_r$, and Δ_{scat} are differences in the imaginary and real part of the polarisability and in the scattering strength along the projected long and short axis of the gold nanorod (see X and Y direction in the main text Fig. 2(a)). For small particles, in which losses are dominated by absorption, and scattering can be neglected, the anisotropy loss tangent Φ is:

$$\Phi = \frac{Im(\alpha'_L - \alpha'_W)}{Re(\alpha'_L - \alpha'_W)} = \frac{Im(\alpha_L - \alpha_W)}{Re(\alpha_L - \alpha_W)} \tag{13}$$

i.e., it is a property of the particle, independent of both the position and orientation of the particle in the cavity mode.

For larger particles, scattering is not negligible. The photon loss rate introduced by Rayleigh scattering is:

$$\Gamma_{sc} = \frac{\sigma_{sc}c}{n_m V_m} I(\vec{r}) = \frac{\omega_c}{2V_m} \frac{8\pi^2 n_m^3 V_p}{3\lambda^3} \alpha^2 I(\vec{r})\beta(\vec{r}) \quad (14)$$

where σ_{sc} is the scattering cross section of the particle, c is the speed of light in vacuo, V_p is the particle volume, and $\beta(\vec{r})$ is a dimensionless factor by which the bulk optical density of states is multiplied at the particle position. Therefore the loss anisotropy parameter in the presence of Rayleigh scattering is given by:

$$2\eta = \frac{\omega_c \Delta\alpha_i}{2V_m} I(\vec{r}) + \frac{\omega_c A}{2V_m} \Delta(\alpha^2) I(\vec{r})\beta(\vec{r}) \quad (15)$$

where $A = \frac{8\pi^2 n_m^3 V_p}{3\lambda^3}$ and $\Delta(\alpha^2)$ is the anisotropy in α^2 for the particle.

The anisotropy loss tangent becomes:

$$\Phi = \frac{Im(\alpha_L - \alpha_W) + A\beta(\vec{r})\{\sin^2\theta\alpha_L^2 - (\cos^2\theta + 1)\alpha_W^2 + 2\cos^2\theta[Re(\alpha_L)Re(\alpha_W) + Im(\alpha_L)Im(\alpha_W)]\}}{Re(\alpha_L - \alpha_W)} \quad (16)$$

It can be seen that in this case, Φ is dependent both on the particle's position $\vec{r}$ and on its polar orientation θ within the cavity frame of reference. The positional dependence comes about because the optical density of states $\beta(\vec{r})$ within the cavity mode is a function of position [6]. The orientation dependence is due to the fact that η is quadratically dependent on the polarizability while ε remains linearly dependent.

To deduce the shape anisotropy from the experimental data in this case, we utilise the histogram of Φ for the single particle event and fit an analytic probability distribution. Assuming a nanorod in free diffusion with no preferred orientation in 3 dimensions, the probability distribution can be expressed as:

$$p(\Phi)d\Phi = p(\theta)d\theta = sin\theta d\theta \quad (17)$$

Differentiating equation (16) then yields:

$$p(\Phi) = \frac{Re(\alpha_L - \alpha_W)}{2A\beta(\vec{r})[\alpha_L^2 + \alpha_W^2 - 2Re(\alpha_L)Re(\alpha_W) - 2Im(\alpha_L)Im(\alpha_W)]B(\Phi)} \quad (18)$$

where

$$B(\Phi) = cos\theta = \sqrt{\frac{\Phi Re(\alpha_L - \alpha_W) - Im(\alpha_L - \alpha_W) - A\beta(\vec{r})(\alpha_L^2 - \alpha_W^2)}{A\beta(\vec{r})[2Re(\alpha_L)Re(\alpha_W) + 2Im(\alpha_L)Im(\alpha_W) - \alpha_L^2 - \alpha_W^2]}} \quad (19)$$

which was used to perform the fitting in Figure 3 of the main text.

Without scattering, we directly used a Gaussian instrumental response to fit the histograms of the anisotropy loss tangent Φ, and obtained the standard error for this fit. Then we used the error propagation equation to calculate the standard error of μ from that of Φ with the equation $SE(\Phi) = \frac{\partial\Phi}{\partial\mu} SE(\mu)$. In the case considering the scattering, we convoluted the probability distribution of the anisotropy loss tangent Φ with the Gaussian instrumental response to perform the fitting. Thus, we employed the bootstrap method and set the percentile range of 0.68 to get the standard error for μ.

7. Spherical Particle Data

To demonstrate that non-zero S_1 signals are introduced by the particle's anisotropy, we measured gold nanospheres of 60 nm diameter (742015-25mL, Sigma-Aldrich) for comparison. The particles are stably suspended in citrate buffer. From the FWHM of S_0 signals over a 500 ms window, increases in photon loss indicating the presence of a particle are visible. Two red-rectangular areas in Figure S3(a) represent two single-particle events. The corresponding S_0, S_1 and S_3 traces over the same 500 ms time window are shown in Figure S3(b), revealing that the S_0 and S_3 peaks both attenuate as a result of this increased loss while no signal is seen in the S_1 trace. Zooming into individual sweeps (Figure S3(c)), confirms this result.

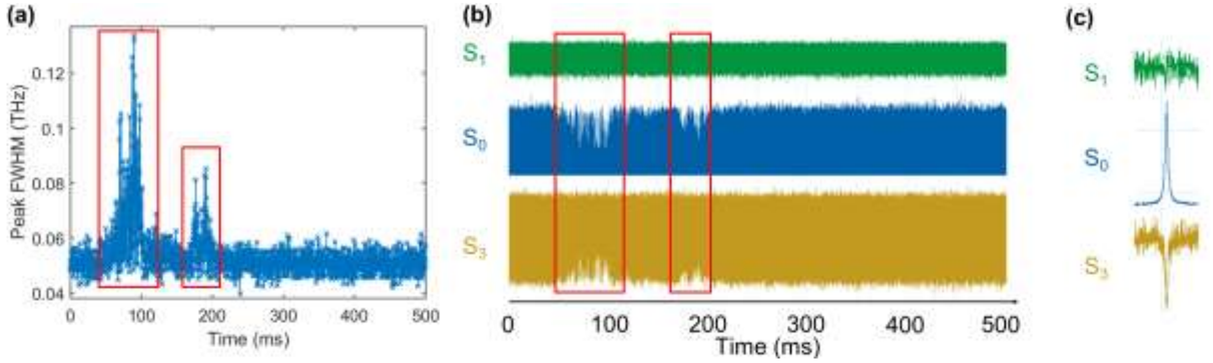

Figure S3: Data of gold nanospheres with a diameter of 60 nm. (a) FWHM of the S_0 peak was measured using the APD over a 500 ms window, showing two discrete single-particle events due to loss-induced broadening. (b) Raw data of S_0, S_1, and S_3 over this 500 ms window. (c) An example of an individual sweep after zooming in.

8. Rotational Diffusion Analysis

There are three common models to derive the rotational diffusion coefficient for rod-shaped particles, with different applicable ranges for the aspect ratio μ, including Perrin theory [7], [8], [9], [10] for $\mu < 2$, Tirado and Garcia de la Torre (TG) theory [11], [12] for $2 < \mu < 30$, and Broersma's relations (BR) [12], [13] for $\mu > 5$. With our gold nanorod in the range of $\mu < 2$, we calculated with Perrin theory (equations 20a and b) and also compared with TG theory (20c). Within Perrin theory, equation (20a) [9] and (20b) [10] produce slightly different values:

$$D_r = \frac{3k_BT}{2\pi\eta L^3}\frac{\mu^4}{\mu^4-1}\left[\frac{(2\mu^2-1)\ln\left(\mu+\sqrt{\mu^2-1}\right)}{\mu\sqrt{\mu^2-1}}-1\right] \tag{20a}$$

$$D_r = \frac{k_BT}{4\pi\eta V}\frac{\mu^2}{\mu^4-1}\left[\frac{(2\mu^2-1)\ln\left(\mu+\sqrt{\mu^2-1}\right)}{\mu\sqrt{\mu^2-1}}-1\right] \tag{20b}$$

$$D_r = \frac{3k_BT}{\pi\eta L^3}\left(ln\mu - 0.662 + \frac{0.917}{\mu} - \frac{0.05}{\mu^2}\right) \tag{20c}$$

where η is the viscosity of the fluid, water in this case, V is the particle size, T is the temperature and k_B is the Boltzmann constant. With the rotational diffusion coefficient, we calculate the averaged angular displacement over $1\ \mu s$ using $<\theta^2>= 2D_rt$. The characteristic angular diffusion time $\tau = \frac{\pi^2}{8D_r}$ is also calculated. The summary is shown in Table S1.

	**Perrin's equation ($\mu < 2$, (20a))**	**Perrin's equation ($\mu < 2$, (20b))**	**TG theory ($2 < \mu < 30$)**
Sample A: D=32 nm, L= 48 nm	2.74×10^4 (13.41°)	2.35×10^4 (12.42°)	1.45×10^4 (9.76°)
Characteristic time	45.03 μs	52.50 μs	85.08 μs
Sample B: D=28 nm, L= 40 nm	4.43×10^4(17.03°)	3.85×10^4(15.90°)	2.35×10^4(12.42°)
Characteristic time	27.85 μs	32.04 μs	52.50 μs

Table S1. Summary of rotational diffusion with different models: rotational diffusion coefficients (the corresponding averaged angular displacement over $1\ \mu s$), and the characteristic time.

Apart from the Brownian rotational diffusion, the particle also experiences the optical torque. We calculated the maximum optical torque M in a Gaussian beam under the experimental condition and calculated the corresponding angular displacement over $1\ \mu s$ using $\theta = \frac{M*t*D_r}{k_B T}$ with the diffusion coefficient calculated from equation (20b). Angular displacements of $0.12°$ and $0.19°$ were found for samples A and B respectively, small enough to be neglected.

9. Thermal Effects

Heating of the nanorod by the optical field could in principle result in a change of shape [14], so we calculated the temperature increase on the surface of the gold nanorod during the resonance as follows:

$$\Delta T = \frac{C_{abs} I}{4\pi R_{eff} \kappa \beta} \tag{21}$$

where C_{abs} is the absorption cross-section, I is the intensity of incident light, R_{eff} is the effective radii if the particle were a sphere, κ is the thermal conductivity of water, and $\beta = 1 + 0.97 \ln^2(\frac{L}{2r})$ is the thermal capacitance coefficient for rod shape of the particle [15]. The absorption cross section is deduced from Gans modification to Mie theory as below [2], [3], [4]:

$$C_{abs} = \frac{2\pi V_p \epsilon_m^{\frac{3}{2}}}{3\lambda} \sum_{x,y,z} \frac{\left(\frac{1}{p_i^2}\right)\epsilon_2}{\{\epsilon_1 + [\frac{1-p_i}{p_i}]\epsilon_m\}^2 + \epsilon_2{}^2} \tag{22}$$

where V_p is the volume of the particle, ϵ_m is the permittivity of the media, ϵ_1, ϵ_2 are the real and imaginary part of the permittivity of gold at the wavelength $\lambda = 640$ nm of the incident light, and p_i $(i = x, y, z)$ are the depolarisation factors along the three axes of the nanorod, as in equation (9). From the APD voltage of the transmitted light from the empty cavity, we calculate the intra-cavity light intensity. The calculated temperature increases for sample A and B are 11K and 7K respectively. These temperature changes are too small to induce any shape change in the nanoparticles.

10. Relationship Between Φ and μ

From equation (8) and (9),

$$\alpha_L - \alpha_W = \frac{\epsilon - \epsilon_m}{\epsilon_m + p_L(\epsilon - \epsilon_m)} - \frac{\epsilon - \epsilon_m}{\epsilon_m + \frac{1-p_L}{2}(\epsilon - \epsilon_m)} \tag{23}$$

Let $\frac{\epsilon}{\epsilon_m} - 1 = \epsilon' - 1 = C + iD$, equation (23) becomes:

$$\alpha_L - \alpha_W = \frac{(C+iD)^2(1-3p_L)}{2+(1+p_L)C+p_L(1-p_L)(C^2-D^2)+iD(1+p_L)+2iCD(1-p_L)p_L} \tag{24}$$

Therefore, the anisotropy loss tangent Φ for the particle becomes:

$$\Phi = \frac{Im(\alpha_L - \alpha_W)}{Re(\alpha_L - \alpha_W)} = \frac{D[4C+(C^2+D^2)+(C^2+D^2)p_L]}{2(C^2-D^2)+[C+Cp_L+(C^2+D^2)p_L-(C^2+D^2)p_L^2](C^2+D^2)} \tag{25}$$

Fig. 3(e) in the main text illustrates this relationship numerically.

11. SEM Results and analysis

The SEM images of gold nanorod samples A and B are illustrated in Figure S4.

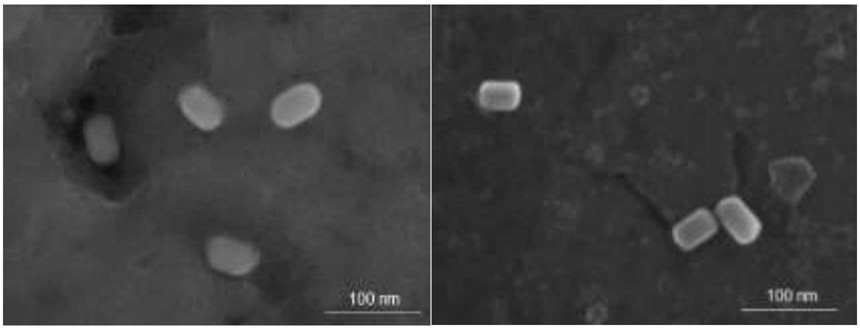

Figure S4. Exemplar SEM images of gold nanorod samples. The left and right hand images correspond to samples A and B respectively.

For a prolate spheroidal nanoparticle of aspect ratio μ oriented with its major axis at an angle θ_S to the normal to the SEM image plane, the aspect ratio measured from the SEM image will be

$$\mu_{SEM} = (\mu - 1)\sin\theta_S + 1 \tag{26}$$

Assuming a random distribution of orientations, $p(\mu_{SEM})d\mu_{SEM} = p(\theta_S)d\theta_S = sin\theta_S d\theta_S$ and the probability distribution of μ_{SEM} is therefore:

$$p(\mu_{SEM}) = \frac{\mu_{SEM} - 1}{(\mu - 1)\sqrt{(\mu - 1)^2 - (\mu_{SEM} - 1)^2}} \tag{27}$$

Convoluting this function with a Gaussian instrumental response function provides an analytic expression which was then fitted to the measured histogram of aspect ratios recorded from SEM image analysis. The fitting results are shown in Figure S5. The fits yield 1.56 ± 0.14 and 1.56 ± 0.18 for samples A and B respectively.

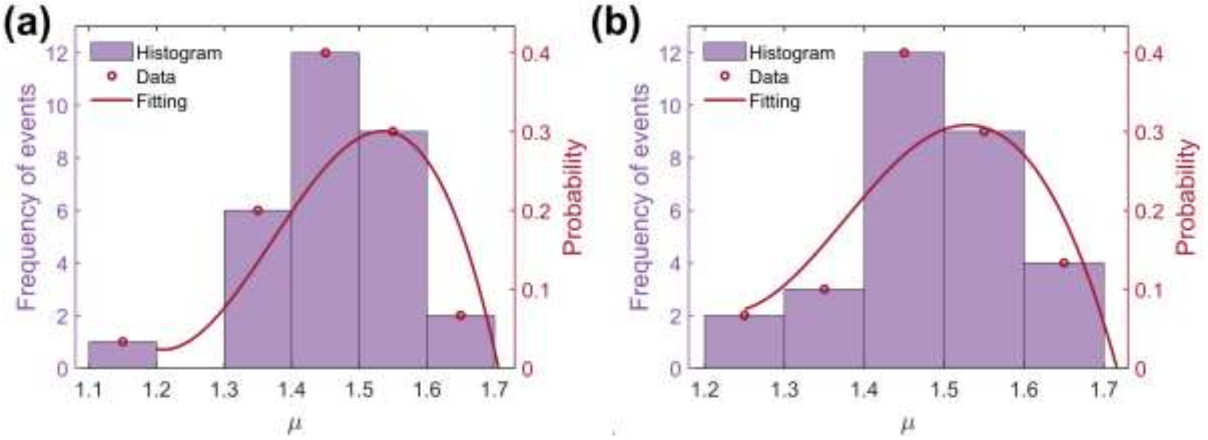

Figure S5: Histograms of particle aspect ratios measured from SEM data, and fits to an analytic function assuming random particle orientations. (a) and (b) are results for samples A and B respectively.

12. Minimum Aspect Ratio Analysis

The particles measured here, with aspect ratios of around $\mu = 1.5$, give signal-to-noise ratios (SNRs) of approximately 7:1 in the S_1 traces shown in Figure 2. In the limit where $\varepsilon, \eta \ll \gamma$, and selecting $\Delta = 0$, equation (7b) reduces to

$$S_1(\varDelta) = \frac{4F^2(\varepsilon sin2\psi - \eta cos2\psi)}{\gamma^3} \tag{28}$$

which is linearly dependent on $\Delta\alpha$ and, in turn, on $\mu - 1$. The aspect ratio that would give SNR = 1 is therefore approximately $\mu = 1.07$.

References

1. Schaefer, B.; Collett, E.; Smyth, R.; Barrett, D.; Fraher, B. Measuring the Stokes polarization parameters. *Am J Phys*. **2007**, *75*(2), 163–168, DOI: 10.1119/1.2386162
2. Gans, R. Über die Form ultramikroskopischer Silberteilchen. *Ann Phys*. **1915**, *352*(10), 270–284, DOI: 10.1002/andp.19153521006
3. Gans, R. Über die Form ultramikroskopischer Goldteilchen. *Ann Phys*. **1912**, *342*(5), 881–900, DOI: 10.1002/andp.19123420503
4. Papavassiliou G. C. Optical properties of small inorganic and organic metal particles. *Progress in Solid State Chemistry*. **1979**, *12*(3–4), 185–271, DOI: 10.1016/0079-6786(79)90001-3
5. Johnson, P. B.; Christy, R. W. Optical Constants of the Noble Metals. *Phys Rev B*. **1972**, *6*(12), 4370–4379, DOI: 10.1103/PhysRevB.6.4370
6. Trichet, A .A. P.; Dolan, P. R.; James, D.; Hughes, G.M.; Vallance, C; Smith, J.M. Nanoparticle trapping and characterization using open microcavities. *Nano Lett*. **2016**, *16*(10), 6172–6177, DOI: 10.1021/acs.nanolett.6b02433
7. Perrin, F. Mouvement brownien d'un ellipsoide - I. Dispersion diélectrique pour des molécules ellipsoidales. *Journal de Physique et le Radium*. **1934**, *5*(10), 497–511, DOI: 10.1051/jphysrad:01934005010049700
8. Koenig, S. H. Brownian motion of an ellipsoid. A correction to Perrin's results. *Biopolymers*. **1975**, *14*(11), 2421–2423, DOI: 10.1002/bip.1975.360141115
9. Kumar, A. H.; Thomson, S. J.; Powers, T. R.; Harris, D. M. Taylor dispersion of elongated rods. *Phys Rev Fluids*. **2021**, *6*(9), 094501, DOI: 10.1103/PhysRevFluids.6.094501
10. Han, Y.; Alsayed, A.; Nobili, M; Yodh, A. G. Quasi-two-dimensional diffusion of single ellipsoids: Aspect ratio and confinement effects. *Phys Rev E*. **2009**, *80*(1), 011403, DOI: 10.1103/PhysRevE.80.011403
11. Tirado, M. M.; De La Torre, J. G. Rotational dynamics of rigid, symmetric top macromolecules. Application to circular cylinders. *J Chem Phys*. **1980**, *73*(4), 1986–1993, DOI: 10.1063/1.440288
12. Nixon-Luke, R.; Bryant, G. A depolarized dynamic light scattering method to calculate translational and rotational diffusion coefficients of nanorods. *Particle & Particle Systems Characterization*. **2019**, *36*(2), 1800388, DOI: 10.1002/ppsc.201800388
13. Broersma, S. Viscous force constant for a closed cylinder. *J Chem Phys*. **1960**, *32*(6), 1632–1635, DOI: 10.1063/1.1730995

14. Chang, S. S.; Shih, C. W.; Chen, C. D.; Lai, W. C.; Wang, C. C. The shape transition of gold nanorods. *Langmuir*. **1999**, *15*(3), 701–709, DOI: 10.1021/la980929l

15. Baffou, G.; Quidant, R.; García de Abajo, F. J. Nanoscale control of optical heating in complex plasmonic systems. *ACS Nano*. **2010**, *4*(2), 709–716, DOI: 10.1021/nn901144d
